# Deep‐dLAMP: Deep Learning‐Enabled Polydisperse Emulsion‐Based Digital Loop‐Mediated Isothermal Amplification

**DOI:** 10.1002/advs.202105450

**Published:** 2022-01-24

**Authors:** Linzhe Chen, Jingyi Ding, Hao Yuan, Chi Chen, Zida Li

**Affiliations:** ^1^ Department of Biomedical Engineering School of Medicine Shenzhen University Shenzhen 518060 China; ^2^ Guangdong Key Laboratory for Biomedical Measurements and Ultrasound Imaging Department of Biomedical Engineering School of Medicine Shenzhen University Shenzhen 518060 China; ^3^ School of Life Sciences and Engineering Southwest Jiaotong University Chengdu Sichuan 610031 China; ^4^ Department of Nanoengineering University of California San Diego La Jolla CA 92093 USA

**Keywords:** deep learning, digital LAMP, digital PCR, nucleic acid test

## Abstract

Digital nucleic acid amplification tests enable absolute quantification of nucleic acids, but the generation of uniform compartments and reading of the fluorescence requires specialized instruments that are costly, limiting their widespread applications. Here, the authors report deep learning‐enabled polydisperse emulsion‐based digital loop‐mediated isothermal amplification (deep‐dLAMP) for label‐free, low‐cost nucleic acid quantification. deep‐dLAMP performs LAMP reaction in polydisperse emulsions and uses a deep learning algorithm to segment and determine the occupancy status of each emulsion in images based on precipitated byproducts. The volume and occupancy data of the emulsions are then used to infer the nucleic acid concentration based on the Poisson distribution. deep‐dLAMP can accurately predict the sizes and occupancy status of each emulsion and provide accurate measurements of nucleic acid concentrations with a limit of detection of 5.6 copies µl^‐1^ and a dynamic range of 37.2 to 11000 copies µl^‐1^. In addition, deep‐dLAMP shows robust performance under various parameters, such as the vortexing time and image qualities. Leveraging the state‐of‐the‐art deep learning models, deep‐dLAMP represents a significant advancement in digital nucleic acid tests by significantly reducing the instrument cost. We envision deep‐dLAMP would be readily adopted by biomedical laboratories and be developed into a point‐of‐care digital nucleic acid test system.

## Graphical abstract

Droplet digital PCR requires sophisticated instruments for droplet generation and reading, limiting their widespread applications. In this article, polydisperse emulsions are generated and read using common instruments, and deep learning is adopted to analyze the occupancy and volumes of the emulsions for nucleic acid quantification.

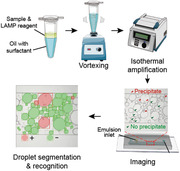

## 1 Introduction

Nucleic acid detection plays a central role in the identification of pathogen infection, diagnosis of genetic diseases, and genetic analysis.^[^ ^1^ ^]^ Nucleic acid amplification test, particularly real‐time polymerase chain reaction (qPCR), has been the primary method for nucleic acid detection. Despite that, the quantification of qPCR requires the reference of standards with known concentrations, which complicates the test and induces inter‐laboratory variations.

Digital PCR (dPCR) provides a powerful means for the absolute quantification of nucleic acids without using references. Droplet digital PCR (ddPCR), a commonly used dPCR method, compartmentalizes the sample into tens of thousands of water‐in‐oil droplets with uniform, pre‐defined volumes and performs PCR therein.^[^ ^2^, ^3^, ^4^, ^5^ ^]^ By enumerating the occupied and empty droplets, the nucleic acid concentration can be inferred using the Poisson distribution. Since the quantification assumes uniform droplet volumes, ddPCR normally uses microfluidics to generate highly monodisperse droplets, making it costly. Though various economical alternatives, such as centrifuge ^[^ ^6^ ^]^ and impact printing,^[^ ^7^ ^]^ have been proposed, the operation could not be easily translated to different laboratories. In terms of droplet reading, a straightforward method is to analyze the fluorescence images of still droplets in a microchamber,^[^ ^8^, ^9^ ^]^ but it is labor‐intensive and difficult to scale up.^[^ ^10^ ^]^ In‐flow interrogation of droplets’ fluorescence offers much higher throughput, but it requires sophisticated devices such as laser and photomultiplier tubes for efficient excitation and signal detection, making the setup complicated and costly. Digital nucleic acid tests with economical, reliable compartmentalization and reading have yet to be achieved.

To simplify the compartmentalization step, the strategy of using non‐uniform volumes has been proposed and demonstrated to be efficacious.^[^ ^11^, ^12^ ^]^ For example, polydisperse droplets were generated using vortexing, and PCR was performed therein. In the reading step, droplet volumes were measured in addition to droplet occupancy,^[^ ^11^ ^]^ and the occupancy and volume data were then used to infer the sample concentration based on the Poisson distribution. In this strategy, droplets could be generated using very convenient means like vortexing,^[^ ^12^, ^13^, ^14^ ^]^ avoiding the need for a costly specialized instrument for uniform droplet generation. Nevertheless, fluorescence imaging‐based reading of droplet occupancy and volumes has yet to be simplified.

Loop‐mediated isothermal amplification (LAMP) generates a byproduct of magnesium pyrophosphate that forms visible precipitates under brightfield, providing a label‐free indicator of the amplification product alternative to fluorescent probes.^[^ ^15^, ^16^ ^]^ However, presumably due to the challenge in image analysis, non‐uniform volume digital LAMP based on in‐flow detection of droplets and precipitates has not yet been reported.

To develop a low‐cost digital nucleic acid test that could be implemented using standard instruments, here we implemented a deep learning‐enabled polydisperse emulsion‐based digital LAMP (deep‐dLAMP) for nucleic acid quantification. The deep‐dLAMP method consists of four major steps, namely emulsion generation and LAMP reaction, emulsion imaging in a flow cell, deep learning image analysis (emulsion segmentation, volume regression, and precipitate‐based occupancy classification), and statistical inference of nucleic acid concentration. Deep‐dLAMP can accurately predict the size and occupancy status of each droplet with a throughput of up to hundreds of droplets per video frame. In the application of deep‐dLAMP, the method provides accurate nucleic acid quantification when the sample concentration ranges from 37.2 to 11000 copies µl^−1^ with a limit of detection of 5.6 copies µl^−1^. We further show that deep‐dLAMP provides robust measurements using different vortexing parameters and cameras with varying image qualities, even when the emulsion brightness and size distributions are not within the deep learning training data distribution. The deep‐dLAMP method developed in this work represents a significant advancement in the nucleic acid detection field by combining the accessible hardware (vortex mixer, thermal cycler, and camera‐coupled brightfield microscope available in most laboratories) and state‐of‐the‐art deep learning models. It can be readily adopted by common biomedical laboratories and opens a new path for developing point‐of‐care digital nucleic acid test systems with significantly reduced instrument costs.

## 2 Experimental Section

### 2.1 Reagents

The LAMP reactions primarily used a commercial kit that detects Proteus mirabilis (051011M, Guangzhou Double Helix Gene Technology Co.). The primer sequences and reagent composition are tabulated in Table S1 and S2, Supporting Information, respectively. Detection samples were prepared by serial dilution of plasmids with target sequences. The DNA concentration of the stock plasmids was (1.12 ± 0.09) × 10^4^ copies µl^−1^, as measured by fluorescence‐based droplet digital LAMP. The detection of SARS‐CoV‐2 used a proprietary primer kit and plasmid (LP1002, HaiGene Biotech Co.), targeting the ORF1ab gene. The sequence of the target gene is tabulated in Table S3, Supporting Information. The continuous phase of the emulsions used a commercial droplet generation oil (1864006, Bio‐Rad Laboratories, Inc.). FITC‐conjugated IgG (A16097, Invitrogen; 0.04 mg ml^−1^) and TRITC‐conjugated IgG (HA1016, HuaBio; 0.01 mg ml^−1^) were used for droplet tracing in the examination of droplet coalescence.

### 2.2 Fabrication of Microfluidic Chips

The microfluidic chips were fabricated using the standard process of SU‐8 photolithography and polydimethylsiloxane (PDMS) replica molding. Fabrication of the SU‐8 masters was outsourced to a microfabrication company (Suzhou Research Materials Microtech Company, China). Upon arrival, the SU‐8 molds were oxygen plasma‐treated (PDC‐002, Harrick Plasma) and exposed to silane vapor in a vacuum chamber for 10 h. PDMS prepolymers (Sylgard 184, Dow, Inc.) with a base‐to‐hardener ratio of 10:1 were mixed, degassed, and poured onto the SU‐8 masters before being baked at 60 °C for 10 h. The cured PDMS was then peeled off and cut into desired shapes before inlets and outlets were punched out. The PDMS slabs and glass slides were treated with oxygen plasma for 1 minute, placed in contact, and baked at 110 °C briefly for bonding. The devices were then baked at 60 °C for 24 h to turn the channel surface hydrophobic.

### 2.3 Emulsion Formation and LAMP Amplification

Polydisperse emulsions were formed using a vortex mixer (LP Vortex Mixer, Thermo Scientific). 50 µl droplet generation oil and 25 µl prepared samples (LAMP mix and DNA samples) pipetted into a 0.2 ml PCR tube (PCR‐0208‐C & PCR‐2CP‐RT‐C, Axygen). Unless otherwise mentioned, the tube was vortexed at 3200 rpm for 15 s. The emulsified samples were then incubated at 63 °C for 45 min using a real‐time PCR instrument (QuantStudio 1, Thermo Fisher Scientific) for quality control during the amplification. Monodisperse emulsions were formed using a flow‐focusing microfluidic chip with the flow driven by a negative pressure (−21.33 kPa relative to 1 atm) at the outlet (Figure S4, Supporting Information). The diameters of the resultant droplets were 46.37 ± 1.64 µm (0.052 nl).

### 2.4 Emulsion Imaging

After amplification, the samples were retrieved by a 1 ml plastic syringe connected with tubing (PE60, Scientific Commodities Inc.). The samples were then manually infused into a microfluidic channel where a stream of spacing oil was maintained by infusing oil at 300 µl h^−1^ using a syringe pump (LSP01‐2A, Longer Precision Pump Co.) to ensure reasonable spacing between emulsions. The microfluidic channel had a width of 800 µm and a height of 46.54 ± 1.31 µm, as measured by cutting through a PDMS slab and imaging under a microscope. The flow of emulsions was imaged in brightfield mainly using an inverted microscope (Eclipse Ti2‐E, Nikon) coupled with a camera (DS‐Qi2, Nikon). Videos were captured at a frame rate of 20 frames per second and lasted for 3–5 min, resulting in about 5400–9000 frames for each video. When studying the dependence of the algorithm on image quality, another two imaging systems were used, including an Eclipse Ts2 inverted microscope (Nikon) coupled with a Phantom VEO‐E310L camera (Vision Research) and an Eclipse Ts2‐FL microscope (Nikon) coupled with a DS‐Fi3 camera (Nikon). Fluorescence of DNA‐intercalating reagents was imaged using the Eclipse Ti2‐E microscope.

### 2.5 Deep Learning‐Based Droplet Quantification

Training data were generated using the LabelMe annotation tool.^[^ ^17^ ^]^ A total 638 image frames were taken from the collected videos at different nucleic acid concentrations. For each image, droplet regions were masked and labeled the droplets in terms of the presence of precipitates. The collected images were randomly split into 9:1 train:test ratios. The Mask R‐CNN was used as the image segmentation model and adapted the Mask R‐CNN code by Abdulla for TensorFlow 2 implementation.^[^ ^18^, ^19^ ^]^ The Resnet‐50 model was chosen as the backbone model. The original video frames were resized from size 1368 × 1368 pixels to 768 × 768 pixels for faster model training. The training region of interest (ROI) per image was 512, and the maximum ground truth instances per image during training were 100. The non‐maximum suppression threshold was set to 0.95 to generate a sufficient amount of proposals. The anchor box sizes were 32, 64, 128, 256, and 512 pixels, with strides being 4, 8, 16, 32, and 64 pixels and width‐length ratios of 0.5, 1, and 2. The maximum number of instances per image during the detection was set to 400.

The stochastic gradient descent (SGD) optimization algorithm was used with a learning rate of 0.0005 in model training. The imgaug image augmentation tool^[^ ^20^ ^]^ was used to execute 0 to 2 of the following augmentations on the fly in a training batch: 1) left‐right flipping of 50% of the images, 2) up‐down flipping of 50% of the images, 3) 50% probability of cropping 0–20% of the height/width, 4) affine transformation by scaling height/width by −30% to 30%, translating x/y by −30% to 30% of width/height, shearing by −4 to 4 degrees, or rotating by −90 to 90 degrees, 5) changing the brightness from 10% to 150% of the original value, and 6) Gaussian blurring with random sigma between 0 and 5.0. The augmentations were found to improve the robustness of the model. The models were trained up to 100 epochs.

### 2.6 Data Analysis

The densities of the mix and oil were about 1 and 1.63 g ml^−1^, respectively. Therefore, the emulsions were subject to significant buoyance in the flow cell. When the emulsions were small, it was assumed that the emulsions did not touch the channel bottom and that the emulsions volumes scaled with the cubic of the imaged equivalent diameter ($2\sqrt{\mathrm{Area}/\pi }$). Uniform microfluidic droplets with volumes ranging from 0.02 to 0.4 nl (determined by assuming spherical shape in an unconfined environment) were generated, imaged the droplets in the flow cell, and performed regression to calibrate the relationship between imaged equivalent diameters and emulsion volumes. When the emulsions were large, it was assumed that the emulsions adopted a cylindrical shape and that the volume could be calculated as the product of the imaged area and channel height. The intersection of these two curves (120 µm) was regarded as the transition point between the two assumptions, as shown in Figure S3, Supporting Information. The volume and occupancy status of each emulsion were used to calculate the sample concentration by solving Equation (2). The equation solving was implemented in Python using Newton iteration as described in a published work.^[^ ^12^ ^]^

The limit of detection was calculated as 3.2 times the standard deviation of the calibrated measurements of blank samples. Dynamic range was calculated as the concentration range of input templates for which the *R^2^* of the linear fitting between log output and log input was larger than 0.98.

### 2.7 Statistical Analysis

Relative fluorescence intensity of droplets was determined by taking the mean grayscale in ImageJ and normalized to the grayscale range. 865 droplets were analyzed for the investigation of the correlation between precipitate presence and fluorescence signal, and more than 1000 droplets were analyzed for the investigation of droplet heating and merging. In the validation experiments using different vortex times, each data point represents a single repeat. In all other cases, data represent mean ± SD with *n* ≥ 3. A two‐sided two‐sample *t*‐test was adopted for hypothesis testing, and significance was defined as *p* ≤ 0.05. Origin (OriginLab Corporation) was used for the statistical analysis.

## 3 Results

### 3.1 Overview of the Method

Standard digital nucleic acid amplification tests use monodisperse droplets, and the concentration, *C*, is estimated following:

(1) $$C=-\ln P^{-}/V$$

where *P^−^* is the fraction of negative droplets and *V* is the volume of individual droplets.

For an individual droplet, the probability of being occupied is exp( − *V* · *C*), and the probability of being empty is 1 − exp( − *V* · *C*), as predicted by the Poisson distribution. When using polydisperse droplets, assume that the observation showed *M* empty droplets, with the volume of each droplet being $V_{i}^{-}$, and *N* occupied droplets, with the volume of each droplet being $V_{j}^{+}$. Using maximum likelihood estimation, which maximizes the likelihood of this particular observation, the concentration, *C*, satisfies^[^ ^11^ ^]^

(2) $$\sum_{i=1}^{M}V_{i}^{-}+\sum_{j=1}^{N}V_{j}^{+}=\sum_{j=1}^{N}\frac{V_{j}^{+}}{\left[1-\exp\left(-V_{j}^{+}\cdot C\right)\right]}$$

Solving Equation (2) gives the estimate of sample concentration, *C*.^[^ ^12^ ^]^

**Figure 1:**
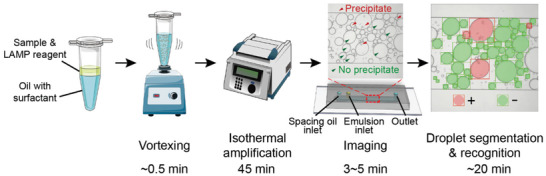
Schematic of the deep learning‐enabled polydisperse emulsion‐based digital loop‐mediated isothermal amplification (deep‐dLAMP). The sample‐reagent mix and oil are vortexed to generate polydisperse emulsions, which then go through isothermal incubation to amplify the nucleic acids of interest, generating precipitate in occupied emulsions. The emulsions are then imaged in a flow cell, and deep learning image analysis is utilized to segment droplets and determine the occupancy based on precipitate presence. The volumes and occupancy status of each emulsion are then used to calculate the nucleic acid concentration. Spacing oil is infused into the flow cell to avoid emulsion packing.

The deep learning‐enabled polydisperse emulsion‐based digital loop‐mediated isothermal amplification (deep‐dLAMP) developed in this work included the following steps. The testing sample and LAMP reagents were mixed, and the mix was emulsified by vortexing using oil with a surfactant as the continuous phase, as shown in **Figure** **1**. The emulsions were then incubated for the LAMP reaction to occur before being imaged in a flow cell. The images were then processed to calculate the volume of each emulsion and classify the emulsion as empty or occupied. LAMP reactions generated the byproduct of magnesium pyrophosphate which was visible as precipitate under bright field.^[^ ^15^ ^]^ Therefore, the presence of precipitates was adopted as the main feature for droplet classification. The processed data were then used to estimate the sample concentration by solving Equation (2).

**Figure 2:**
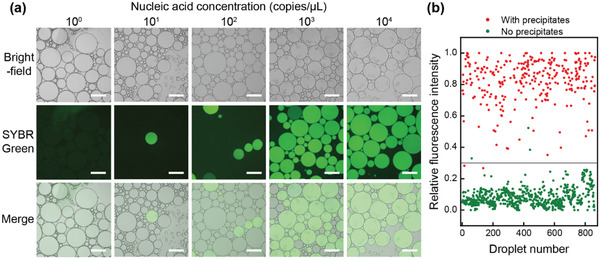
Presence of precipitates as a reliable indicator of occupied emulsions. a) Brightfield, SYBR Green fluorescence, and merged micrographs of emulsions after LAMP reaction using samples with different nucleic acid concentrations. Scale bars, 200 µm. b) Relative fluorescence intensity and precipitate status of each droplet. The presence of precipitate coincided with high fluorescence intensity.

A prerequisite of the successful implementation of deep‐dLAMP is that precipitates can reliably indicate occupied emulsions. We generated emulsions using samples with different nucleic acid concentrations, performed the LAMP reaction, and observed the correlation between the precipitate presence and fluorescence. Results showed that higher nucleic acid concentrations led to a more frequent presence of emulsions with precipitates (**Figure** **2**). If we thresholded the fluorescence intensity at 0.3, 99.0% of the “fluorescently” positive droplets had precipitate (true positive), and 99.6% of the fluorescently negative droplets had no precipitates (true negative), showing that precipitates can indeed serve as a marker of occupied droplets.

Volume change of the droplets, such as evaporation and coalescence during the amplification, poses a challenge to the efficacy of deep‐dLAMP. To characterize the evaporation and coalescence of droplets, we generated uniform droplets of FITC solution and TRITC solution, respectively, using microfluidics (Figure S1, Supporting Information). The two sets of droplets were pooled and incubated at 63 °C for 45 min. The droplet diameters before and after incubation were 54.3 ± 2.2 µm and 54.5 ± 2.0 µm, respectively, with no significant difference, as shown in Figure S2, Supporting Information, suggesting that volume shrinking during the amplification was negligible. This seemingly surprising observation was likely attributed to the heated lid used by the PCR instrument, which prevented a continuous mass transport from the aqueous phase to the tube lid. In addition, results showed that the fluorescence of FITC and TRICT were mostly isolated in individual droplets (Figure S3a, Supporting Information), with most droplets showing either high TRICT or high FITC (Figure S3b, Supporting Information). For example, with a FITC threshold of 0.5 and a TRITC threshold of 0.3, 99.46% of the droplets would fall into the two quadrants with high FITC or high TRITC intensity, suggesting that droplet coalescence was minimal.

### 3.2 Image Analysis Pipeline for Droplet Segmentation and Classification

**Figure 3:**
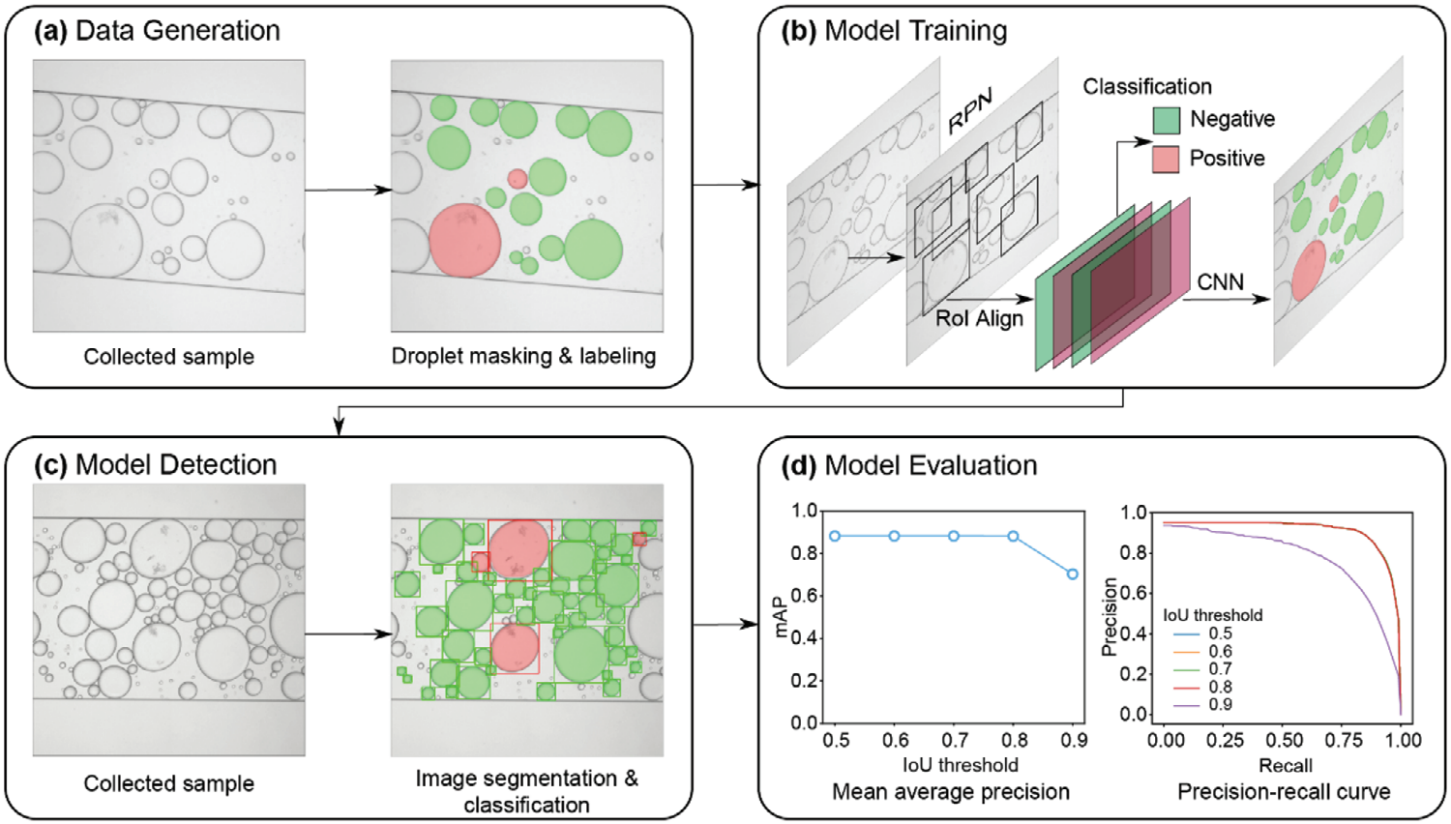
Schematic showing the flow of the deep learning‐enabled droplet segmentation and classification. a) Data generation via droplet area masking/segmentation and occupancy labeling. b) The model training uses the region proposal network (RPN) to scan the image for droplet occurrence. The regions of interest (RoI) are aligned for subsequent classification and droplet region refinement prediction using a convolutional neural network (CNN). c) Model predictions on new samples. Both the droplet regions and occupancy labels are predicted in one shot. d) Model evaluation on the test data set. (left) The mean average precision (mAP) is plotted as a function of the intersection over union (IoU) and (right) the precision‐recall curve as a function of IoU.

We then sought to develop an image analysis pipeline to output the area and occupancy status of each droplet within the frame. We took video frames as static images and then segmented the droplet areas and labeled the corresponding occupancy information manually to generate the initial training data for the model. The image‐to‐label process is shown in **Figure** **3**a, where the “positive” droplets that have precipitates inside were masked in red, while the empty ones or the “negative” droplets were in green. The data label was repeated on 638 video frames with nucleic acid concentrations ranging from 1 to 10^4^ copies µl^−1^. The original images and the associated label and mask information formed the training (90%) and test (10%) data for the Mask R‐CNN model. The Mask R‐CNN scanned each image for droplets using the region proposal networks (RPN). Then regions that contained droplets were aligned to be classified in terms of occupancy information. The exact boundaries of the droplet were predicted using a convolutional neural network (CNN), as shown in Figure 3b. More details about the model implementation and training are in the Methods section. Once the model was trained, it was automatically applied to new images/videos for droplet segmentation and classification (Figure 3c). The mean average precision (mAP) of the model prediction on the test data at different thresholds of intersection over union (IoU) and the precision‐recall curve of the classification were shown in Figure 3d. The trained Mask R‐CNN showed remarkable accuracy with 88% mAP with IoU up to 0.8. When IoU went to 0.9 (the predicted box had to overlap with the true box for more than 90%), the mAP was reduced due to the stringent requirements. The precision‐recall curve also followed the same trend, where a moderate to high (0.5–0.8) IoU threshold gave both high precision and recall.

### 3.3 Analysis of the Measurements

The image analysis pipeline generated two types of data of each emulsion, namely the occupancy status and the droplet area, for the inference of nucleic acid concentrations based on Equation (2). We first performed additional experiments to characterize the relationship between the droplet volume and the measured droplet area (Figure S4; Methods, Supporting Information), and then we designed experiments to validate these two types of data separately.

**Figure 4:**
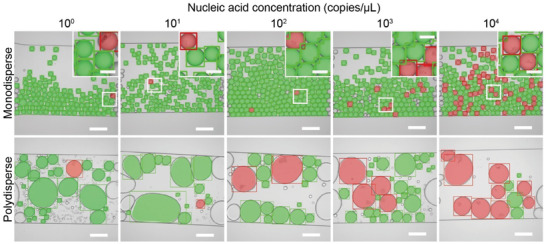
Representative micrographs showing the results of droplet segmentation and classification after the image analysis using monodisperse and polydisperse emulsions on samples with different nucleic acid concentrations. Transparent shadows represent the detected droplet regions, and rectangles represent the bounding boxes, with red indicating the occupied droplets and green the empty droplets. Scale bars, 200 µm in the main figures and 50 µm in the insets.

To validate the detection results of occupancy status, we generated monodisperse droplets with well‐defined sizes (diameter, 46.5 ± 1.3 µm; volume, 0.052 nl) using microfluidics and performed LAMP reaction and the image analysis. After the image analysis, the majority of the droplets were precisely segmented and correctly classified, as demonstrated in **Figure** **4**. The occupancy data were then used to calculate the nucleic acid concentrations using uniform volume assumption following Equation (1). Results showed that at high concentrations, the calculated concentrations were very close to the true concentrations. For example, when the sample concentration was 1.12 × 10^2^, 1.12 × 10^3^, and 1.12 × 10^4^ copies µl^−1^, where the theoretical fractions of positive droplets were 0.57%, 5.5%, and 43.5%, respectively, the calculated concentration was (1.80 ± 0.22) × 10^2^, (0.98 ± 0.04) × 10^3^, and (0.86 ± 0.01) × 10^4^ copies/µl^−1^, respectively, suggesting that the detection accuracy was relatively adequate at these concentrations. When the concentration was lower than 1.12 × 10^2^ copies µl^−1^, the method tended to overestimate the concentration. This inaccuracy was likely because the fractions of positive droplets at these concentrations were very low (0.005% ‐ 0.05%), and that false positives, induced by debris, double emulsion, and dust on the channel walls, could have a dramatic effect on the calculation.

**Figure 5:**
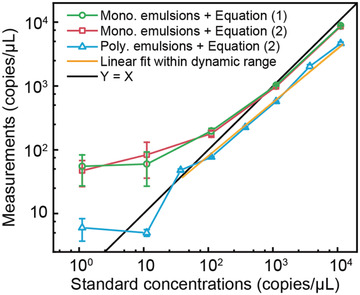
The deep‐dLAMP measurements using monodisperse emulsions calculated by Equation (1) (uniform volume assumption), using monodisperse emulsions calculated by Equation (2) (non‐uniform volume assumption), and using polydisperse emulsions calculated by Equation (2) (non‐uniform volume assumption). The line of *Y* = *X* is shown for reference. Data represent mean ± SD with *n* = 3. Mono., monodisperse. Poly., polydisperse.

To provide a preliminary validation of the volume detection and the algorithm based on non‐uniform volume assumption, we further calculated the nucleic acid concentrations by solving Equation (2). As shown in **Figure** **5**, Equation (2), along with the analyzed volume data, generated very similar results compared to those based on uniform assumption, with relative differences smaller than 10% when the concentration ranged from 10^2^ to 10^4^ copies µl^−1^. At lower concentrations, the differences were around 20 copies µl^−1^. These results suggested that the area detection was accurate and that the algorithm based on non‐uniform assumption could generate comparable measurements with the standard algorithm based on the uniform volume assumption.

We then generated polydisperse droplets by vortexing and performed the complete test and analysis. As demonstrated in Figure 4, most droplets were accurately segmented and classified using the image analysis pipeline. However, three types of droplets were not detected. Droplets that were small, typically with diameters smaller than 24 µm (0.007 nl), required very small boxes for scanning and were computationally expensive to be detected. Droplets that were very large, typically with equivalent diameters larger than 250 µm (8.2 nl), tended to be highly deformed with irregular shapes, making them difficult to be detected. As such, these two types of droplets were left out. Since these volumes accounted for only 2.08% of the total volume and the presence of nucleic acids in each emulsion can be regarded as independent incidence, we believe leaving out these types of droplets would not bring in bias to the measurement. In addition, droplets close to the frame edges with incomplete shapes were not detected by design.

The volume and occupancy data generated from the deep learning image analysis were then used to calculate nucleic acid concentrations based on Equation (2). As shown in Figure 5, when the nucleic acid concentrations were as low as 1.1 copies µl^−1^, the measured concentrations showed suboptimal accuracy, likely due to the unmet high demand on occupancy detection at these concentrations. At higher concentrations, the measured concentrations were much closer to the expected values. For example, the measured concentrations for 1.1 × 10^2^, 1.1 × 10^3^, and 1.1 × 10^4^ copies µl^−1^ were (0.77 ± 0.02) × 10^2^, (0.57 ± 0.01) × 10^3^, and (0.46 ± 0.02) × 10^4^ copies µl^−1^, respectively. Though the measured data showed a systematical underestimation, the logarithm of the measured concentrations showed good linearity with the logarithm of the standard concentrations. In the concentration range of 37.2 to 1.1 × 10^4^ copies µl^−1^, the *R* ^2^ of the linear fitting was 0.99, indicating that the dynamic range spanned from 37.2 to 1.1 × 10^4^ copies µl^−1^. This linear fitting also provided a calibration curve for future measurements, which followed

(3) $$C_{\mathrm{cal}.}=0.50\cdot {C_{\mathrm{mea}.}}^{1.19}$$

where *C*_cal._ is the calibrated measurement and *C*_mea._ is the calculated measurement from Equation (2). The difference between the measured and true concentrations was likely due to the error when converting emulsion area to volume, especially at high concentrations. We additionally performed deep‐dLAMP on three blank samples, and the measurements were 5.24, 5.78, 2.49 copies µl^−1^, respectively, indicating that the limit of detection was 5.6 copies µl^−1^ (see Methods, Supporting Information).

### 3.4 Characterization of the deep‐dLAMP

**Figure 6:**
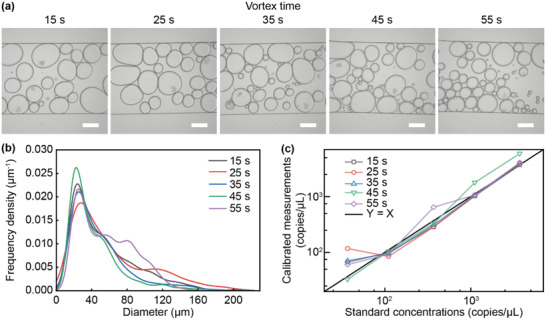
Effect of vortex times on the deep‐dLAMP measurements. a) Representative micrographs of the emulsions in the flow cell generated by different vortex times. Scale bars, 200 µm. b) Frequency densities are plotted as a function of the equivalent diameters of the emulsions generated using different vortex times. c) Calibrated measurements using the standards of different concentrations with different vortex times. The line of *Y* = *X* is shown for reference.

We then sought to investigate how different parameters would affect the deep‐dLAMP measurements. The vortexing processing determined the size distribution of the resultant emulsions and could directly affect the measured concentrations. To examine that, we subjected samples of different nucleic acid concentrations (37.2 ‐ 3720 copies µl^−1^) to a range of vortexing time (15 ‐ 55 s) and performed deep‐dLAMP. As anticipated, the generated emulsions became smaller as the vortex time increased, and the diameter distribution showed a trend of left shift at higher vortex time, as shown in **Figure** **6**a,b. However, the nucleic acid concentrations calculated using deep‐dLAMP did not show a significant dependence on the vortex times. After calibration using Equation (3), measurements from experiment groups using different vortex times mostly had similar values, which were close to the true concentrations, as shown in Figure 6c. To assess the agreement of the calibrated measurements with true values, we calculated the *R^2^* of the calibrated measurements over *Y* = *X* in the log‐log plot. Results showed that the *R^2^* was 0.96, 0.86, 0.96, 0.97, and 0.95 for the vortex time of 15s, 25s, 35s, 45s, and 55s, suggesting that the calibrated measurements could represent the true values. Nevertheless, when the vortex time was high, such as 45 s and 55 s, the calibrated measurements were sometimes higher than expected, which were likely because extended vortexing resulted in more double emulsions that could be falsely identified as occupied emulsions, thus overestimating the concentrations. These results suggested that the deep‐dLAMP measurements were relatively robust on different emulsification conditions, though prolonged vortexing should be avoided.

**Figure 7:**
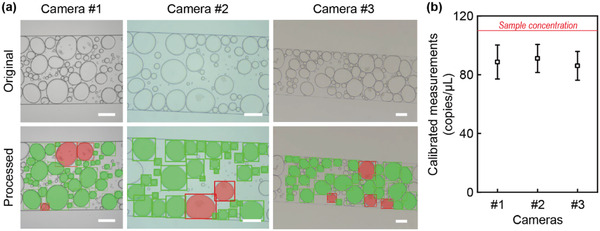
Effect of image qualities on the deep‐dLAMP measurements. a) Representative original micrographs captured by different cameras and the corresponding analyzed images. Scale bars, 200 µm. b) The calibrated measurements using different cameras. Data represent mean ± SD with *n* = 3.

Image quality could have a significant impact on the performance of the droplet segmentation and classification, consequently affecting the measurements. During the model training, we used data augmentation techniques, such as flipping, blurring, transformation, and brightness adjustment, to mimic situations that were different from our training data. To verify the robustness of the model, we performed deep‐dLAMP using two additional microscopes and cameras with different lighting and image qualities. We designated the original camera and these two additional cameras as Camera #1, Camera #2, and Camera #3, respectively. As demonstrated in **Figure** **7**a, the image analysis pipeline was able to accurately segment and classify emulsions. In addition, the calibrated measurements from Camera #2 and Camera #3 were 91.0 ± 9.6 and 86.0 ± 9.8 copies µl^−1^, respectively, both of which were not significantly different from that of 88.6 ± 11.6 copies µl^−1^ from Camera #1 (Figure 7b). These results suggested that the deep‐dLAMP can generate robust measurements in different laboratories equipped with different microscopes and cameras.

The specificity of deep‐dLAMP mainly depends on the performance of the LAMP primers and the amplification reactions. As such, the specificity of deep‐dLAMP should be as good as the LAMP reaction itself. Nonetheless, we performed experiments to verify this point. We used two primer sets, which were designed to detect SARS‐CoV‐2 and Proteus mirabilis, respectively, and cross‐tested the corresponding samples using the deep‐dLAMP. As shown in Figure S6, Supporting Information, little cross‐reaction was observed, suggesting that the deep‐dLAMP had good specificity.

## 4 Discussion and Conclusions

In this work, we reported deep learning‐enabled polydisperse emulsion‐based digital loop‐mediated isothermal amplification (deep‐dLAMP) to perform digital nucleic acid amplification tests using easily accessible laboratory equipment. In deep‐dLAMP, polydisperse emulsions were generated to perform LAMP reaction therein, and the precipitates of magnesium pyrophosphate were detected by deep learning‐based image analysis to determine emulsion occupancy. The volume and occupancy of each emulsion were then used to calculate the nucleic acid concentration based on the Poisson distribution. We further showed that deep‐dLAMP measurements were robust at different vortex times and image qualities. Compared with commercial droplet digital PCR, deep‐dLAMP holds the merits of low instrument cost and easy operation (Table S4, Supporting Information). We envision that deep‐dLAMP can serve as a low‐cost, portable nucleic acid test with absolute quantification.

In the current form, deep‐dLAMP was implemented by capturing images at 20 frames per second, and each frame was regarded as an independent sampling in the data analysis. As such, emulsions were repetitively sampled for the concentration calculations. Though we performed analysis to justify this sampling strategy, as shown in Figure S6, Supporting Information, it would be desirable to perform droplet tracking in the videos. Consequently, each droplet would be counted only once in the concentration inference, and the overall detected volume could be explicitly reported. In addition, as each droplet would be detected and classified in a few frames, the occupancy status could be decided by the majority vote, which could potentially further improve the detection and measurement accuracy.

Using the calibration curve of Equation 3, the calibrated results showed robustness on different experimental conditions, including different samples and flow chambers. It suggested that the calibration mainly corrected the inaccurate droplet volumes in the calculation, and deep‐dLAMP still provides an absolute quantification as long as the volume data is properly corrected. Therefore, if the design of the flow chambers is consistent, repeated calibration would not be necessary. Nevertheless, in future work, it is desirable to confirm that inaccurate prediction of droplet volumes indeed contributed to the underestimation of the nucleic acid concentration. For example, droplet volumes may be measured more accurately using sophisticated 3D imaging technologies. In terms of the experimental operation, the current procedure of droplet imaging requires a syringe pump and manual emulsion injection since a spacing oil is required to separate emulsions apart and maintain their round shapes. Though this fluidic strategy served the purpose of validating the concept of the deep‐dLAMP, it would be beneficial to develop more user‐friendly droplet reading methods in the future. For example, by designing a negative pressure‐driven flow chamber with separate inlets of spacing oil and emulsion, the reading step can be achieved by simply pulling an empty syringe at the outlet. In addition, using oil (such as mineral oil) with a similar density as water could potentially eliminate the need for spacing oil and further simplify the droplet reading setup.

## Conflict of Interest

The authors declare no conflict of interest.

## Authors Contribution

L.C. and J.D. contributed equally to this work. Z.L. conceptualized the project. L.C., J.D., and H.Y. performed the experiments. C.C. developed the image analysis pipeline. L.C., J.D., C.C., and Z.L. analyzed the data. L.C., C.C., and Z.L. wrote the manuscript. All authors contributed to the manuscript.

## Supporting information

Supporting Information

Supplemental Video 1

Supplemental Video 2

## Data Availability

The data that support the findings of this study are available from the corresponding author upon reasonable request.
