# Supplementary material for: Deep‐dLAMP: Deep Learning‐Enabled Polydisperse Emulsion‐Based Digital Loop‐Mediated Isothermal Amplification
Source: Adv Sci (Weinh). 2022 Jan 24;9(9):2105450. doi: 10.1002/advs.202105450 (PMC8948574; doi:10.1002/advs.202105450)
Supplement: Supplementary file 1 — Supporting Information [file ADVS-9-2105450-s002.pdf]

ADVANCED SCIENCE
Open Access

Supporting Information

for *Adv. Sci.*, DOI 10.1002/advs.202105450

Deep-dLAMP: Deep Learning-Enabled Polydisperse Emulsion-Based Digital Loop-Mediated Isothermal Amplification

Linzhe Chen, Jingyi Ding, Hao Yuan, Chi Chen and Zida Li**

ADVANCED SCIENCE
Open Access

Supporting Information

for *Adv. Sci.*, DOI: 10.1002/advs.202105450

deep-dLAMP: deep learning-enabled polydisperse emulsion-based digital loop-mediated isothermal amplification

Linzhe Chen, Jingyi Ding, Hao Yuan, Chi Chen, and Zida Li**

Supplementary Materials

deep-dLAMP: deep learning-enabled polydisperse emulsion-based digital loop-mediated isothermal amplification

Linzhe Chen[1,2,#], Jingyi Ding[1,2,#], Hao Yuan[3], Chi Chen[4,], and Zida Li[1,2,*]*

[1]Department of Biomedical Engineering, School of Medicine, Shenzhen University, Shenzhen 518060, China;

[2]Guangdong Key Laboratory for Biomedical Measurements and Ultrasound Imaging, Department of Biomedical Engineering, School of Medicine, Shenzhen University, Shenzhen 518060, China;

[3]School of Life Sciences and Engineering, Southwest Jiaotong University, Chengdu, Sichuan 610031, China;

[4]Department of Nanoengineering, University of California San Diego, La Jolla, CA, 92093, USA.

[#]Contributed equally.

[*]Correspondences: chc273@eng.ucsd.edu; zidali@szu.edu.cn

Supplementary Tables

Table S1: Primer sequences (5'-3') for the detection of Proteus mirabills.

**FIP**	CGGCAATTAATAGCTCACGGTA-GCAAGCTGAAATTGAAGAACG
**BIP**	CCAACTGACCTGCTTGAAGCT-GTATGAATATTTAGCAGGACGT
**F3**	TTAATGAACGCCGCGAAG
**B3**	TCCAAGTTTTAGTTTCACCATT
**LoopB**	GCTGGCGCAAGCAAAACTG
**Target Gene**	TTAATGAACGCCGCGAAGAAGAACAAGCAATGCA-AGCTGAAATTGAAGAACGTCAGCAAAAATTACAAA-AATACCGTGAGCTATTAATTGCCGATGGTATTGATCC-AACTGACCTGCTTGAAGCTGCTGGCGCAAGCAAAACT-GGTCGTGCTAAACGTGCAGCACGTCCTGCTAAATATTC-ATACGTTGATGATAATGGTGAAACTAAAACTTGGA

Table S2. Reagents for a 25 μL premix.

**Reagent**		**Volume/μL**	**Final concentration**
Reaction mix	dNTP	12.5	2.8 mM
	Tris-HCl		40 mM
	KCl		20 mM
	$MgSO_4$		16 mM
	$(NH4)_2SO_4$		20 mM
	Betaine		1.6 M
Bst DNA polymerase		1	8 U/μL
Primers	FIB/BIP	1	40 μM
	F3/B3		5 μM
	LoopB		20 μM
SYBR-Green I		0.5	-
Template		10	1.1 ~ 11,000 copies/μL

Table S3: Target sequence (5’-3’) in the detection of SARS-CoV-2.

**ORF1ab Gene**	AACAGTGGTTGTTAATGCAGCCAATGTTTACCTTAAACAT-<br>GGAGGAGGTGTTGCAGGAGCCTTAAATAAGGCTACTAAC-<br>AATGCCATGCAAGTTGAATCTGATGATTACATAGCTACTA-<br>ATGGACCACTTAAAGTGGGTGGTAGTTGTGTTTTAAGCGG-<br>ACACAATCTTGCTAAACACTGTCTTCATGTTGTCGGCCCAA-<br>ATGTTAACAAAGGTGAAGACATTCAACTTCTTAAGAGTGC-<br>TTATGAAAATTTTAATCAGCACGAAGTTCTACTTGCACCAT-<br>TATTATCAGCTGGTATTTTTGGTGCTGACCCTAT

Table S4: Comparison with Bio-Rad QX200.

	**deep-dLAMP**	**Bio-Rad QX200**
Instrument cost	~ 5 k USD	~120 k USD[*]
Consumable cost (cartridge and oil)	~$2 per test<br>(~$1 for cartridge;<br>~$1 for oil)	~$5 per test[*]<br>(~$3 for cartridge;<br>~$2 for oil)
Droplet generation time	~0.5 minutes	~2.5 minutes[**]
Amplification time	45 minutes	40 minutes[*]
Reading time	3-5 minutes	~12.5 minutes[*]
Complete workflow time	1.5-2 hours	4.5-5.5 hours[***]
Dynamic range	37.2-1.1×10^4 copies/μL	5×10^{-2}-6×10^3 copies/μL[**]

[*]Based on experience.

[**]Based on *Digital Droplet PCR Applications Guide* published by Bio-Rad Laboratories, Inc.

[***]Based on *QX200 Droplet Reader and QuantaSoft Software Instruction Manual* published by Bio-Rad Laboratories, Inc.

Supplementary Figures

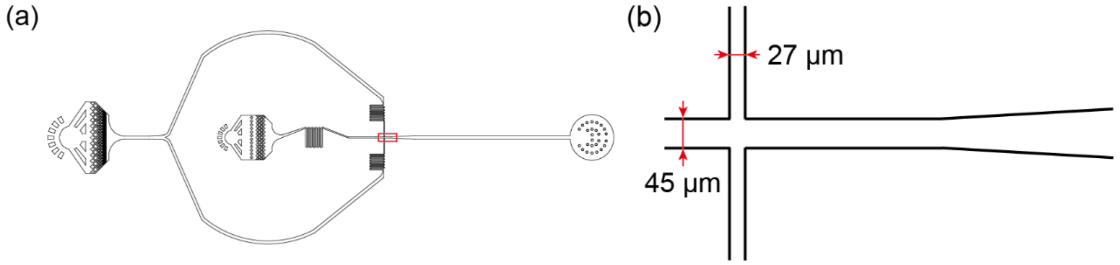

Figure S1. (a) The design of the microfluidic chip for the generation of uniform emulsions. (b) Close-up of the red-boxed area in Panel (a).

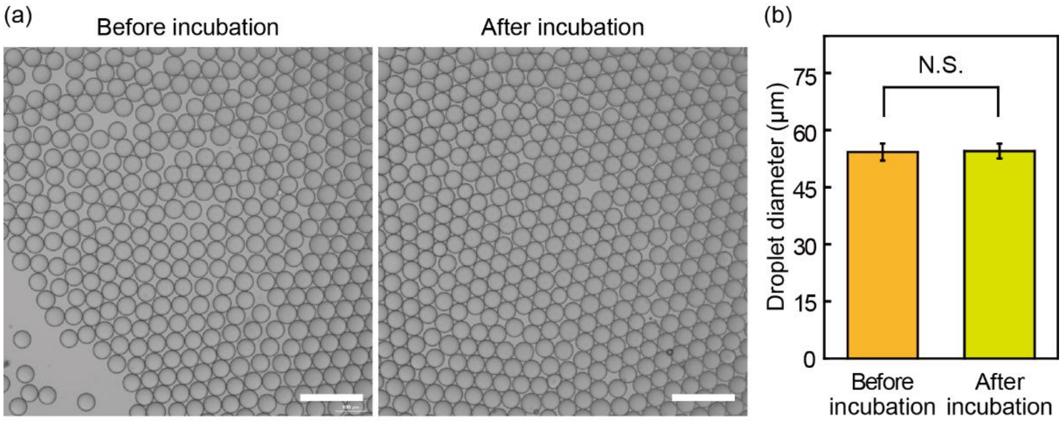

Figure S2. Droplet evaporation during incubation is negligible. (a) Representative micrographs of droplets before and after incubation. Scale bars, 200 μm. (b) Bar plot of droplet diameter before and after incubation. Data represent mean ± SD with n > 1000. N.S., P > 0.05.

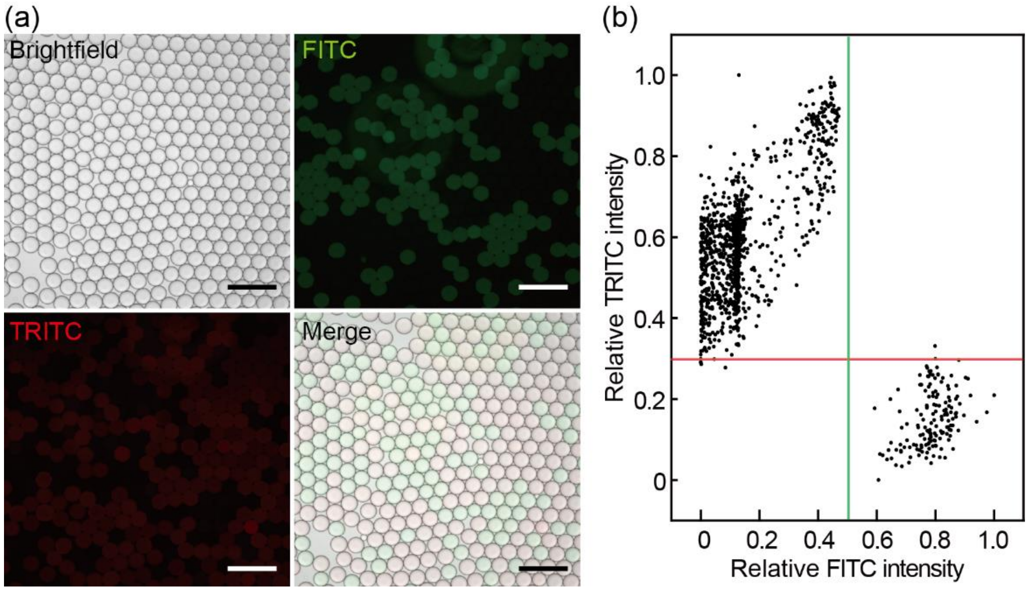

Figure S3. Droplet coalescence during incubation is negligible. (a) Brightfield, FITC, TRITC, and merged micrographs of the mixture of FITC droplets and TRITC droplets after incubation. FITC droplets and TRITC droplets were separately generated and pooled before incubation. Scale bars, 200 μm. (b) Scatter plot of the relative FITC and TRITC intensity of each droplet. Red and green lines indicate a threshold of 0.3 and 0.5, respectively.

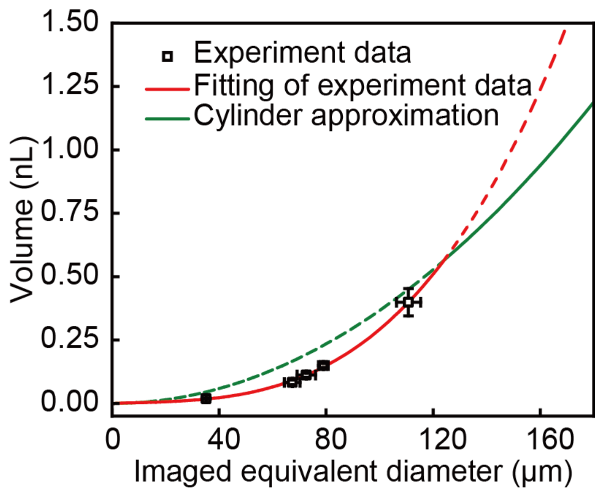

Figure S4. Calibration of the relationship between the volumes and the imaged equivalent diameters of the emulsions in the flow cell. The imaged equivalent diameter was defined as $2\sqrt{Area/\pi}$. Experiment data were obtained by generating uniform emulsions, which were loaded into the flow cell to determine the imaged equivalent diameters and placed on a cover slip to determine the true volume. The experiment data were fitted by assuming that the volumes scaled with the cubic of the imaged equivalent diameter. When emulsions were large, this assumption did not hold any longer, and the volumes were calculated assuming a cylinder shape. Cylinder approximation calculated the volumes as $\pi d^2 h/4$, where d was the imaged equivalent diameter and h was the channel height (~46 μm).

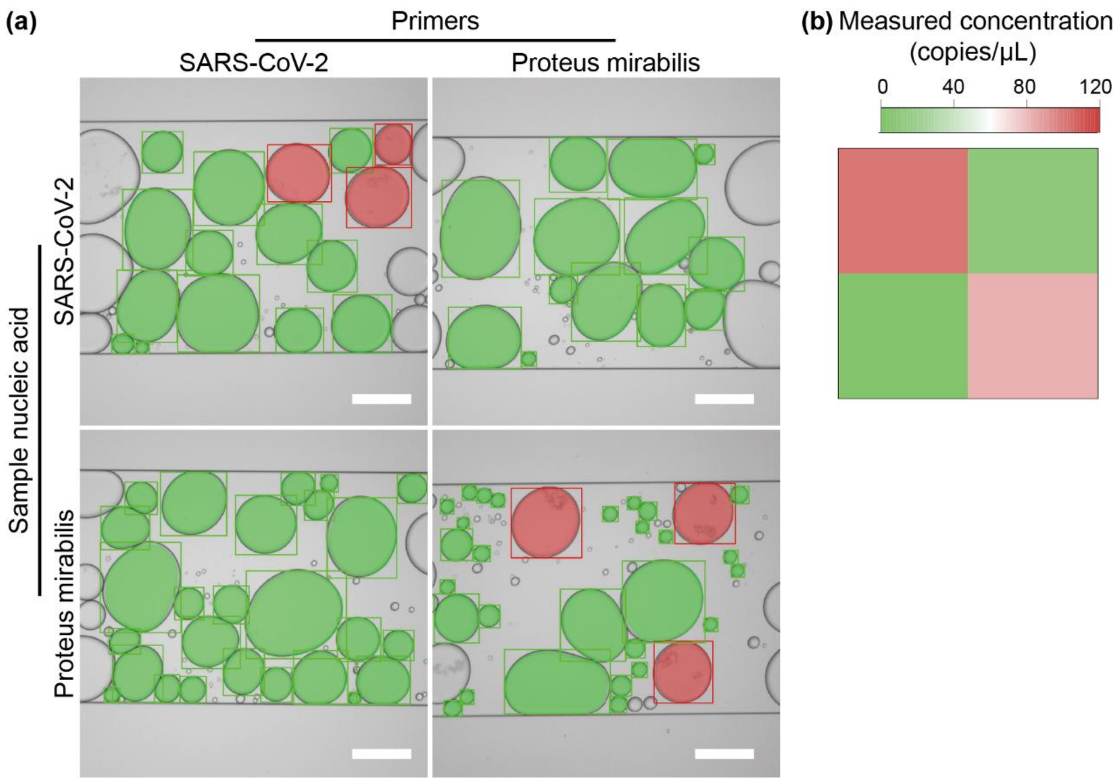

Figure S5. Specificity of the deep-dLAMP measurements. (a) Representative processed micrographs of emulsions using the combinations of two sets of primers and two samples, relevant to SARS-CoV-2 and Proteus mirabilis nucleic acid, respectively. Scale bars, 200 μm. (b) Heat map showing the measured nucleic acid concentrations of the four sample/primers combinations placed corresponding to the arrangement in Panel (a).

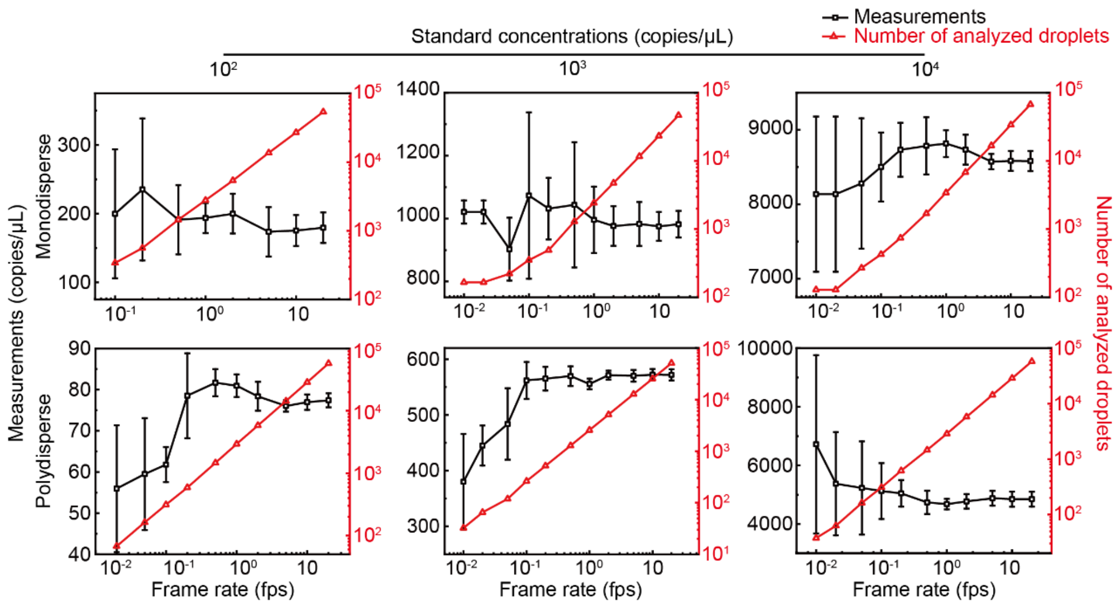

Figure S6. Effect of frame sample rates on the deep-dLAMP measurements on samples of different concentrations using monodisperse and polydisperse emulsions, respectively. Original videos were taken at 20 fps, and different frame rates in this analysis were achieved by downsampling the frames from the original videos. When the frame rates were higher than 1 fps, no significant changes in measurements were observed, indicating that the strategy of counting individual emulsions repetitively was valid.

Video S1. Representative video showing the droplet segmentation and classification using monodisperse emulsions.

Video S2. Representative video showing the droplet segmentation and classification using polydisperse emulsions.
